# Epithelial Cancer of the Lips and Face

**Published:** 1877-09-20

**Authors:** 


### EPITHELIAL CANCER OF THE LIPS AND FACE.

Professor Busch, of Bonn, in a communication to the Congress of German Surgeons, began by stating the theory ot Thiersch, according to which this form of cancer originates in a disturbance of the equilibrium between the epidermis and the connective tissue. Cancer of the scrotum and of the breast would also come under this head. The majority of such cancers occur in the face and lips. The disease originates in an induration, which grows from the surface inwards, often no notice being taken of it at the commencement. It looks at first quite harmless, and is, especially on the lips, scarcely visible when it commences. Chronic cases begin with the growth of a horny substance on the skin. When this is removed, a bleeding surface is found. After removing this with the blade of a knife, a number of roots are seen penetrating into the skin. Still deeper, small scales of epidermis are discovered pierced by the epithelium roots, which penetrate deeper and deeper into the tissues. This progress is mostly observed on the skin of the aged, and the origin of the disease is very often proven to be of an external, perhaps of a chemical nature. Whether the presence of such a layer of horny substance is alone sufficient to produce cancer, is a question not yet settled. After removing this horny substance by softening it with a solution of soda, and then keeping the place well moistened and clean with the same fluid, the slight excavation will be seen to disappear, and even the epidermis will grow again. Professor Volkman some time ago pointed out the favorable influence of cleanliness in similar affections.

Professor Busch removes at first the horny substance, and, after the extirpation of the cancer, directs that the spot and its surroundings should be well washed with the solution of soda. He lays great stress upon this, for, on operating on even the worst cases of this form of cancer, one can scarcely tell which are the sound and which are the diseased portions. A patient, who had been cured of rodent ulcer, discontinued these washings after a year. But a few months later the scab reappeared, and under it the swollen surface. Another case is still more instructive. After extirpation of a cancer, the washings were soon neglected. Immediately afterwards a new scab formed on the lip. Washing was commenced again, and again discontinued. Finally, a new extirpation became necessary. Washing of the lips with the alkaline lotion is efficacious only at the beginning of a case; it has been observed to be of the greatest service in cases of rodent ulcer of the face. There can be no doubt that the moist morphœa and many other diseases of the skin are to be considered as the beginnings of epithelioma. Of special importance in this class of cases is the cancer in the breasts of elderly women. Deposits of horny substance are very often observed, and beneath them enlarged milk-vessels; and it is extremely likely that cancer of the mamma very often takes its origin from these deposits.

Where there is hereditary predisposition, Professor Busch recommends extirpation of such deposits, which are often apt to cause injurious pressure. He has observed the development of cancer out of simply a horny deposit. The disease begins with the obstruction of the milk-vessels by epithelial masses, and its progress is to be arrested by preventing these obstructions from taking place. Professor Busch mentioned a case in which thick layers of the epidermis covered the nipple. After disolving them with a solution of soda, he succeeded, by using slight pressure, in removing from the openings of the milk-canals diminutive white plugs, consisting of epithelium in a state of fatty degeneration. This treatment was continued for three months, and during this time an induration that had some time previously been discovered in the breast also disappeared.

Such success, however, is to be expected only in recent cases. Professor Busch's communication concluded with the following propositions: 1. The beginning of a destructive epithelial cancer is in many cases a simple epithelial deposit on the surface. 2. The disease in this state is to be cured by continued local treatment (washing with the soda solution). 3. This treatment may also be successful in some favorable cases of carcinoma of the skin of the face, even where ulcers already exist. 4. In many cases recurrence after extirpation may be prevented by washing the scar and its surroundings with alkaline lotion. 5. A very desirable precaution is the removal of the layers of epithelium that are sometimes to be found on the breasts of elderly females.—*Med. Examiner*, April 26, 1877.

